# *EGFR* ‐plasma mutations in prognosis for non‐small cell lung cancer treated with EGFR TKIs: A meta‐analysis

**DOI:** 10.1002/cnr2.1544

**Published:** 2021-08-23

**Authors:** Thang Thanh Phan, Vinh Thanh Tran, Bich‐Thu Tran, Toan Trong Ho, Suong Phuoc Pho, Anh Tuan Le, Vu Thuong Le, Hang Thuy Nguyen, Son Truong Nguyen

**Affiliations:** ^1^ The Laboratory D Unit Clinical Cancer Center, Cho Ray Hospital Ho Chi Minh City Vietnam; ^2^ Faculty of Biology‐Biotechnology University of Science, VNU‐HCM Ho Chi Minh City Vietnam; ^3^ Department of Chemo‐Radiotherapy Clinical Cancer Center, Cho Ray Hospital Ho Chi Minh City Vietnam; ^4^ Department of Thoracic Disease Cho Ray Hospital Ho Chi Minh City Vietnam; ^5^ Department of Clinical Pathology Cho Ray Hospital Ho Chi Minh City Vietnam; ^6^ Department of the Vice Minister Ministry of Health Hanoi City Vietnam

**Keywords:** ctDNA, *EGFR*, NSCLC, prognosis

## Abstract

**Background:**

The plasma‐based epidermal growth factor receptor (*EGFR*) mutation testing is approved recently to use in clinical practice. However, it has not been used as a prognostic marker yet because of contradictory results.

**Aim:**

This meta‐analysis aims to clarify the role of the *EGFR*‐plasma test in prognosis for non‐small cell lung cancer (NSCLC) who have mutant tumors and receive EGFR tyrosine kinase inhibitors (TKIs).

**Methods and Results:**

The PubMed/MEDLINE, Web of Science, Cochrane Library, and Google Scholar databases were searched for relevant studies by April 10, 2021. The hazard ratio (HR) from reports was extracted and used to assess the correlation of *EGFR*‐plasma status with progression‐free survival (PFS) and overall survival (OS). A total of 35 eligible studies with 4106 patients were enrolled in the final analysis. Patients with concurrent *EGFR* mutations in pretreatment plasma have shorter PFS (HR = 2.00, 95% confidence interval [CI]: 1.73–2.31, *p* < .001) and OS time (HR = 2.31, 95% CI: 1.89–2.83, *p* < .001) compared to the tumor‐only mutation cases. Besides, the persistence of *EGFR‐*activating mutations in post‐treatment plasma is associated with worse PFS (HR = 3.84, 95% CI: 2.96–4.99, *p* < .001) and OS outcome (HR = 3.22, 95% CI: 2.35–4.42, *p* < .001) compared to others. Notably, the prognostic value of the *EGFR*‐plasma test is also validated in treatment with third‐generation EGFR TKI and significance regardless of different detection methods.

**Conclusion:**

The presence of *EGFR*‐plasma mutations at pretreatment and after EGFR TKI initiation is the worse prognostic factor for PFS and OS in NSCLC.

## 1 BACKGROUND

EGFR TKIs have been recommended as the first‐line agents in treatment for NSCLC patients for many years.^1^ Accordingly, biopsy procedures must be done to get tumor tissues, then tested for the drug sensitivity mutations as *EGFR* ^E19del^ (exon 19 deletions) and *EGFR* ^L858R^ (Leucine‐to‐Arginine point mutation in exon 21). Unfortunately, not all patients are eligible for biopsy procedures, while the failure rate of biopsy might be high as 20%, accompanied by dangerous complications.^2^ In such cases, *EGFR* mutation testing in plasma samples is an alternative method that assists the initial diagnosis and also helps in treatment monitoring. Although the *EGFR*‐plasma test is approved to use in clinical practice recently,^1^ it has not been used as a prognostic marker yet because of contradictory results.^3^, ^4^, ^5^, ^6^, ^7^, ^8^, ^9^, ^10^, ^11^, ^12^, ^13^, ^14^ In meta‐analyses of Mao C and Fan G,^3^, ^4^ authors concluded that patients with *EGFR* mutations in the blood are associated with improved PFS and OS outcomes, which are different from the evidence of recent clinical trials.^5^, ^6^, ^7^, ^8^, ^9^, ^10^, ^11^, ^12^, ^13^, ^14^ These analyses were conducted on studies that included both *EGFR*‐positive and *EGFR*‐negative patients.^3^, ^4^ Currently, *EGFR*‐negative patients are not introduced to treatment with EGFR TKIs,^1^ and therefore should not include them in such analyses.^3^, ^4^ Our meta‐analysis aims to clarify the prognostic role of the *EGFR*‐plasma test in mutant tumor NSCLC treated with EGFR TKIs.

## 2 MATERIALS AND METHODS

This meta‐analysis was conducted according to the guideline of preferred reporting items for systematic reviews and meta‐analyses (PRISMA).^15^

### 2.1 Database searching and selection of study

**FIGURE 1:**
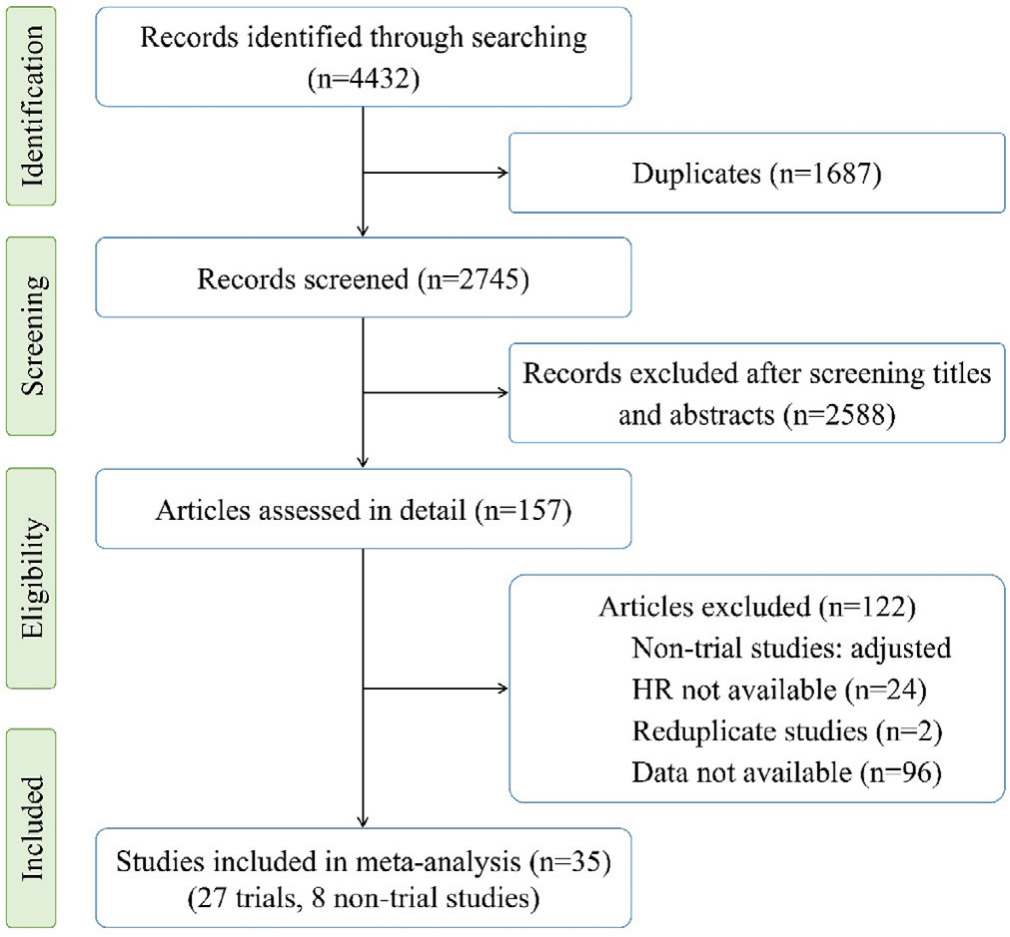
Database searching and study selection

The electronic database as PubMed/MEDLINE, Web of Science, and Cochrane Library were searched for relevant studies. The keywords used in searching include “EGFR,” “ctDNA or circulating tumor DNA,” “cfDNA or circulating free DNA,” “plasma or peripheral blood,” “NSCLC or non‐small cell lung cancer,” “lung cancer,” “lung carcinoma,” “survival,” “outcome,” “PFS,” and “OS.” Besides the above databases, Google Scholar was used for study searching. Moreover, the citation reports of potential studies were also reviewed for finding additional articles. The cut‐off date of database searching is April 10, 2021 (the start date was not applied). After searching, all relevant studies were exported into the EndNote list (4432 records) and removed duplicates (1687 records, Figure 1).

By screening titles and abstracts, 2588 records were excluded from the study, while 157 remained articles were assessed in detail for eligibility. Studies included in the meta‐analysis which are clinical trials meet criteria: (1) dealt with non‐small cell lung cancer who have *EGFR‐*activating mutations (*EGFR* ^E19del^ and *EGFR* ^L858R^ ± *EGFR* ^T790M^) in tumor tissue and treated with EGFR TKIs as gefitinib, erlotinib, icotinib, afatinib, and osimertinib (first‐line and second‐line); (2) analyzed the association of *EGFR* status in paired tumor tissue and plasma/serum (T + P+: *EGFR*+ in both tumor tissue and plasma/serum; T + P‐: *EGFR*+ in tumor tissue but not in plasma/serum) with survival (PFS, OS); (3) have at least five patients in each comparison arms; and (4) have enough information to determine HR directly or indirectly. For the non‐trial studies, besides these criteria, the adjusted HR values must be available. Finally, 35 studies were included in this meta‐analysis (27 clinical trials and 8 non‐trial studies).

### 2.2 Quality assessment and data extraction

The Newcastle‐Ottawa Scale (NOS), which comprises three aspects equivalent to a maximum score of 9 points (selection: 4 points; comparability: 2 points; and outcome: 3 points),^16^ was used to assess the included studies. In the comparability aspect, studies were scored 2 points if (1) comparable of treatment agents, and (2) comparable of patient's characteristics (age, gender, histology, clinical stage, and metastasis status) between two arms (T + P+ and T + P‐).

We extracted data from articles including author's name, publication year, country, study design, the number of patients in each arm, patient's age, clinical stage, sample type, sampling time‐point, the technique used to detect *EGFR* mutations, treatment agent, length of follow‐up, outcome (PFS, OS), HR value, method of survival analysis (univariate/multivariate), and NOS score. In cases of not availability, HR values were calculated indirectly according to the recommendations of Tierney JF.^17^

### 2.3 Statistical analysis

Data analyses were done with the guidance of Harrer,^18^ performed with R statistical software v.4.0.5 (R foundation, 1020 Vienna, Austria), and meta, metafor, dmetar packages. The random‐effects model was used to calculate the pooled HR values and assess the association of *EGFR* plasma status with survival outcomes. HR > 1 indicates an inferior survival for the patients with T + P+ mutations. In contrast, HR < 1 is the indicator of superior survival for T + P+ subjects. HR = 1 suggests that no correlations exist between *EGFR* plasma mutations and survival outcomes.

The heterogeneity of effect size (HR) between studies was measured by Higgin's and Thompson's *I* ^2^‐statistics. Heterogeneity was determined as significant if *I* ^2^ > 50% and *p* < .05. Accordingly, the subgroup analyses were performed to explore sources of heterogeneity that may come from clinical characteristics. Furthermore, we used the Leave‐one‐out statistic to detect studies with extreme effect sizes (outliers). Then, the pooled HR was estimated once removed outliers from the analysis and checked for the consistency of overall results. The potential of publication bias in the meta‐analysis was detected by the linear regression test for funnel plot asymmetry. In case of significant bias presence (*p* < .05), we used the Trim‐and‐fill method to impute missing studies and calculate the adjusted HR values.

## 3 RESULTS

### 3.1 Study characteristics

Among 35 studies included in this meta‐analysis,^5^, ^6^, ^7^, ^8^, ^9^, ^10^, ^11^, ^12^, ^13^, ^14^, ^19^, ^20^, ^21^, ^22^, ^23^, ^24^, ^25^, ^26^, ^27^, ^28^, ^29^, ^30^, ^31^, ^32^, ^33^, ^34^, ^35^, ^36^, ^37^, ^38^, ^39^, ^40^, ^41^ 21 studies reported the association of *EGFR* mutations in prior‐treatment plasma (prior‐*EGFR*) with survival outcomes, including seven studies for PFS, three and 11 studies for OS, and both survival outcomes (Additional file 1: Table S1). Twenty‐two studies presented data related to the post‐treatment *EGFR*‐plasma mutations (post‐*EGFR*), which consists of 11 reports for PFS, one for OS, and 10 for both outcomes. The total number in prior‐treatment studies is 2483 patients, and in post‐treatment are 1623 cases. Ten studies used osimertinib in NSCLC treatment (two with first‐line and eight with second‐line), while others used the first‐ or second‐generation EGFR TKIs with/without chemotherapy. The polymerase chain reaction (PCR) methods were used in almost all studies, while the next‐generation sequencing (NGS) technique was only used in four reports to detect *EGFR‐*plasma mutations. The NOS score above six indicated that all included studies are of high quality.

### 3.2 Association of prior‐treatment *EGFR* plasma with survival outcomes

**FIGURE 2:**
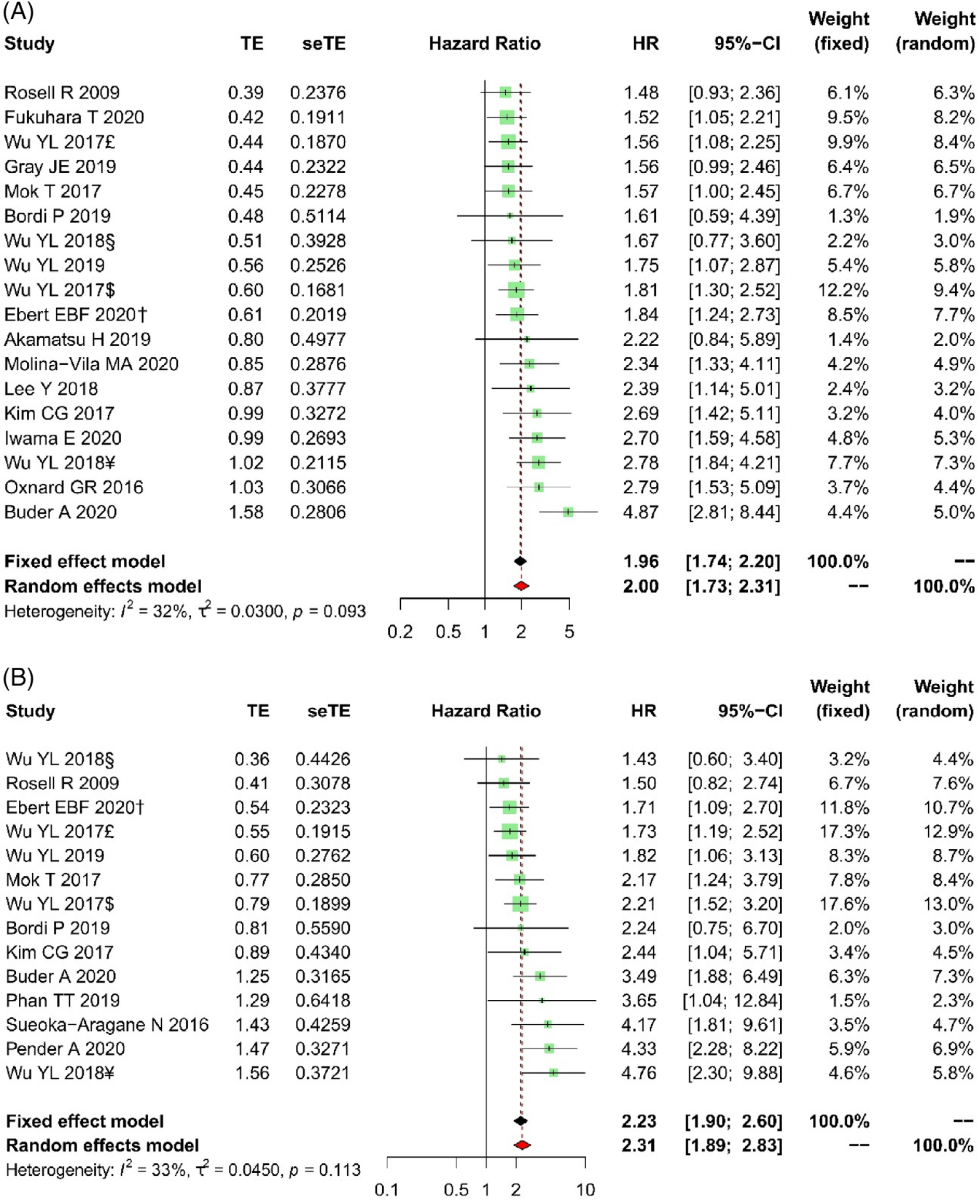
Forest plots of HR for the impact of prior‐*EGFR* on PFS (A) and OS (B). HR, hazard ratio; OS, overall survival; PFS, progression‐free survival

**FIGURE 3:**
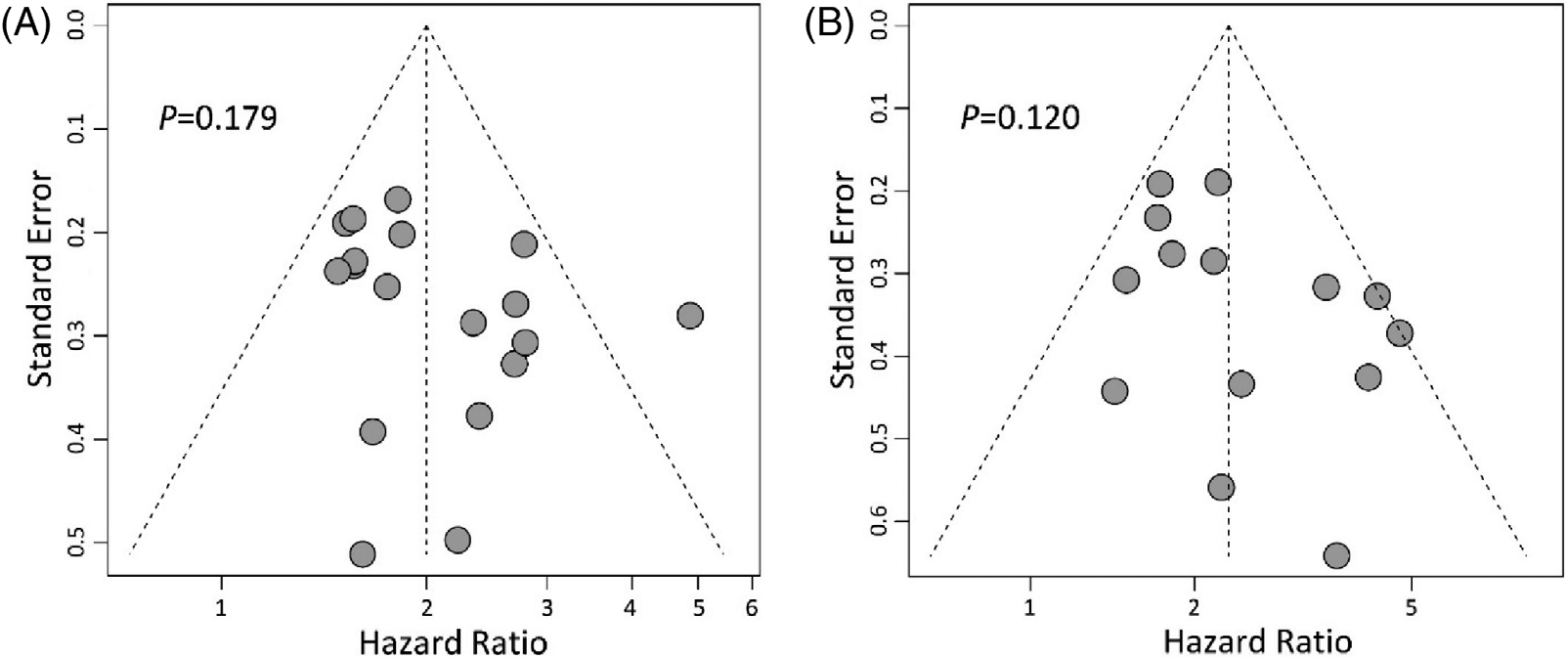
Funnel plots for publication bias in analyses with prior‐*EGFR* for PFS (A) and OS (B). OS, overall survival; PFS, progression‐free survival

Among 2483 patients in the studies with prior‐*EGFR*, 1524 patients have the T + P+ *EGFR* mutations, whereas 959 others have the T + P‐ results. The PFS time of T + P+ patients was from 3.7 to 15.6 months, and of T + P‐ subjects were 8.3 months to “not reached” (NR). These OS values were 8.2–28.8 months and 25.3–NR months, respectively. The overall estimated HR for PFS was 2.00 (95% CI: 1.73–2.31, *p* < .001, Figure 2A), which indicated that *EGFR*+ in both tumor tissue and plasma at baseline is the worse prognostic factor for NSCLC treated with EGFR TKIs. Similarly, the analysis has shown that T + P+ *EGFR* mutation is the inferior factor for OS (HR = 2.31, 95%CI: 1.89–2.83, *p* < .001, Figure 2B). The heterogeneity in these analyses for PFS (*I* ^2^ = 32%, *p* = .093) and OS (*I* ^2^ = 33%, *p* = .113) were not statistically significant. Besides, funnel plot asymmetry tests indicated a lack of publication bias in these analyses (Figure 3A,B).

The subgroup analysis results for PFS and OS are presented in Tables 1 and 2, respectively. Although significant heterogeneity exists in some subgroups (Caucasian, osimertinib treatment, and non‐clinical trials), overall effect sizes are not significantly different between them (*p* > .05).

**TABLE 1:** Subgroup meta‐analyses of prior‐*EGFR* for PFS

Variable	No. of study	No. of patient	HR (95%CI)	*p*‐Value^*^	Heterogeneity		*p*‐Value^***^
					*I* ^2^, %	*p*‐Value^**^	
Ethnicity							
Asian	9	1001	2.02 (1.72–2.39)	<0.001	0	0.472	0.371
Caucasian	5	543	2.21 (1.45–3.38)	<0.001	67	0.018	
Mixed	4	642	1.69 (1.35–2.12)	<0.001	3	0.379	
Treatment							
1st/2nd‐gen TKI	14	1693	1.88 (1.65–2.13)	<0.001	0	0.559	0.349
Osimertinib	4	493	2.49 (1.40–4.43)	0.002	72	0.014	
Technique							
asPCR	7	1044	1.83 (1.56–2.15)	<0.001	0	0.520	0.143
dPCR	4	285	2.94 (1.86–4.63)	<0.001	39	0.176	
PCR clamping	3	470	1.65 (1.27–2.14)	<0.001	0	0.393	
Other^a^	4	387	2.14 (1.59–2.89)	<0.001	6	0.364	
HR extraction method							
Direct	7	748	2.40 (1.79–3.21)	<0.001	52	0.053	0.086
Indirect	11	1438	1.80 (1.56–2.07)	<0.001	0	0.566	
Survival analysis							
Multivariate	5	522	2.53 (1.65–3.86)	<0.001	62	0.031	0.167
Univariate	13	1664	1.85 (1.62–2.10)	<0.001	0	0.550	
Clinical trial							
No	4	431	2.59 (1.49–4.50)	<0.001	72	0.014	0.262
Yes	14	1755	1.87 (1.65–2.12)	<0.001	0	0.578	

*Note*: ^*^Significance within groups; ^**^significance of heterogeneity; ^***^significance between groups.

^a^ BEAMing, PANAMutyper, MBP‐QP; 1st‐/2nd‐gen: first‐/second‐generation.

Abbreviations: HR, hazard ratio; NGS, next‐generation sequencing; OS, overall survival; PCR, polymerase chain reaction; TKI, tyrosine kinase inhibitor.

**TABLE 2:** Subgroup meta‐analyses of prior‐*EGFR* for OS

Variable	No. of study	No. of patient	HR (95% CI)	*p*‐Value^*^	Heterogeneity		*p*‐Value^***^
					*I* ^2^, %	*p*‐Value^**^	
Ethnicity							
Asian	7	710	2.50 (1.85–3.38)	<0.001	26	0.233	0.372
Caucasian	5	629	2.40 (1.56–3.69)	<0.001	56	0.061	
Mixed	2	295	1.86 (1.36–2.54)	<0.001	0	0.509	
Treatment							
1st‐/2nd‐gen TKI	12	1488	2.24 (1.81–2.78)	<0.001	36	0.103	0.195
Osimertinib	2	146	3.13 (1.83–5.38)	<0.001	0	0.490	
Technique							
ARMS	1	33	3.65 (1.04–12.84)	0.044	‐	‐	0.056
asPCR	6	801	2.01 (1.56–2.58)	<0.001	33	0.192	
dPCR	3	323	3.58 (2.37–5.41)	<0.001	0	0.592	
PCR clamping	1	164	1.50 (0.82–2.74)	0.188	‐	‐	
Other^a^	3	313	2.60 (1.73–3.92)	<0.001	0	0.437	
HR extraction method							
Direct	6	726	2.49 (1.70–3.64)	<0.001	47	0.090	0.577
Indirect	8	908	2.21 (1.74–2.81)	<0.001	26	0.218	
Survival analysis							
Multivariate	5	584	2.80 (1.83–4.29)	<0.001	40	0.156	0.125
Univariate	9	1050	2.12 (1.71–2.62)	<0.001	23	0.242	
Clinical trial							
No	5	584	2.80 (1.83–4.29)	<0.001	40	0.156	0.125
Yes	9	1050	2.12 (1.71–2.62)	<0.001	23	0.242	

*Note*: ^*^Significance within groups; ^**^significance of heterogeneity; ^***^significance between groups.

^a^ BEAMing, PANAMutyper, MBP‐QP; 1st/2nd‐gen: first−/second‐generation.

Abbreviations: HR, hazard ratio; NGS, next‐generation sequencing; OS, overall survival; PCR, polymerase chain reaction; TKI, tyrosine kinase inhibitor.

### 3.3 Association of post‐treatment *EGFR* plasma with survival outcomes

**FIGURE 4:**
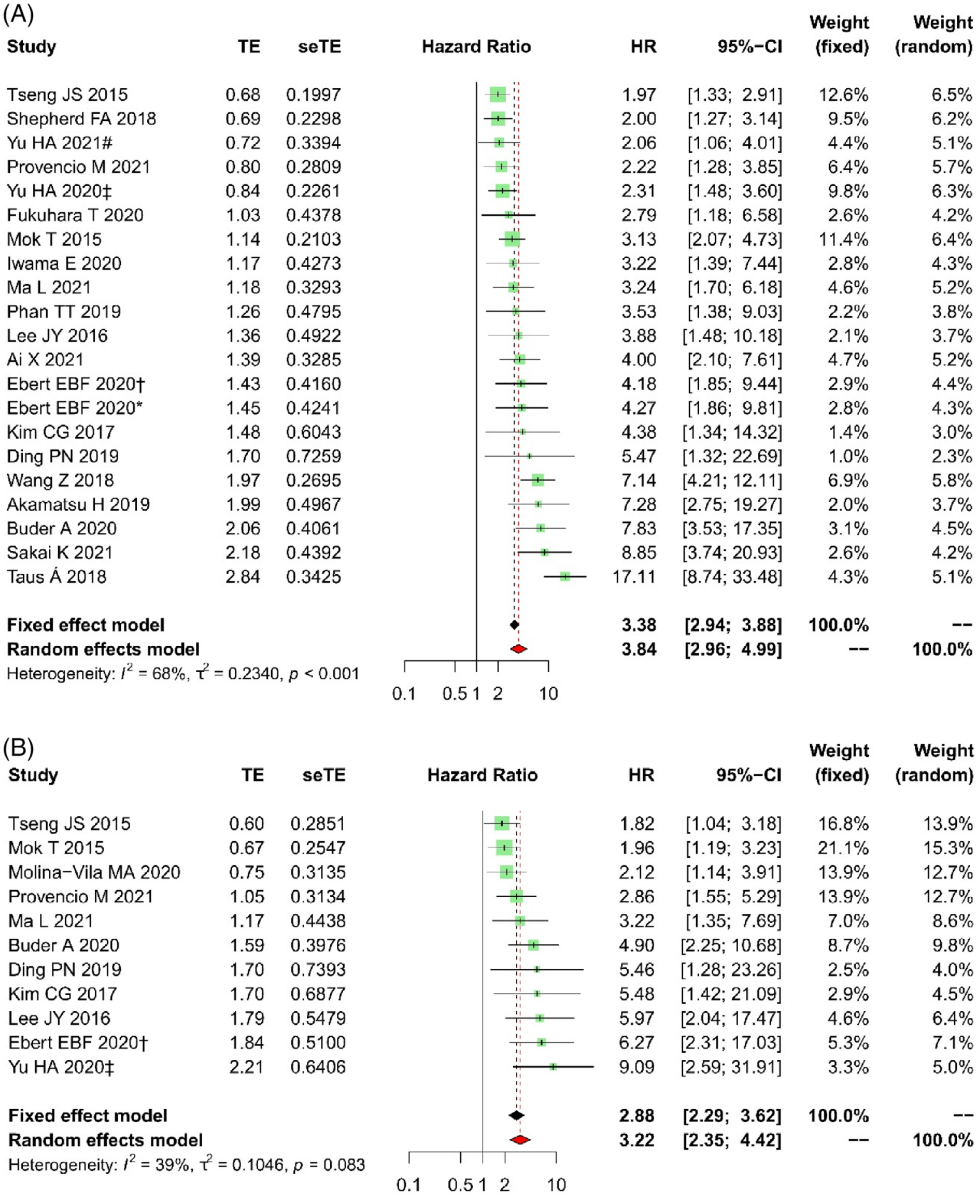
Forest plots of HR for the impact of post‐*EGFR* on PFS (A) and OS (B). HR, hazard ratio; OS, overall survival; PFS, progression‐free survival

**FIGURE 5:**
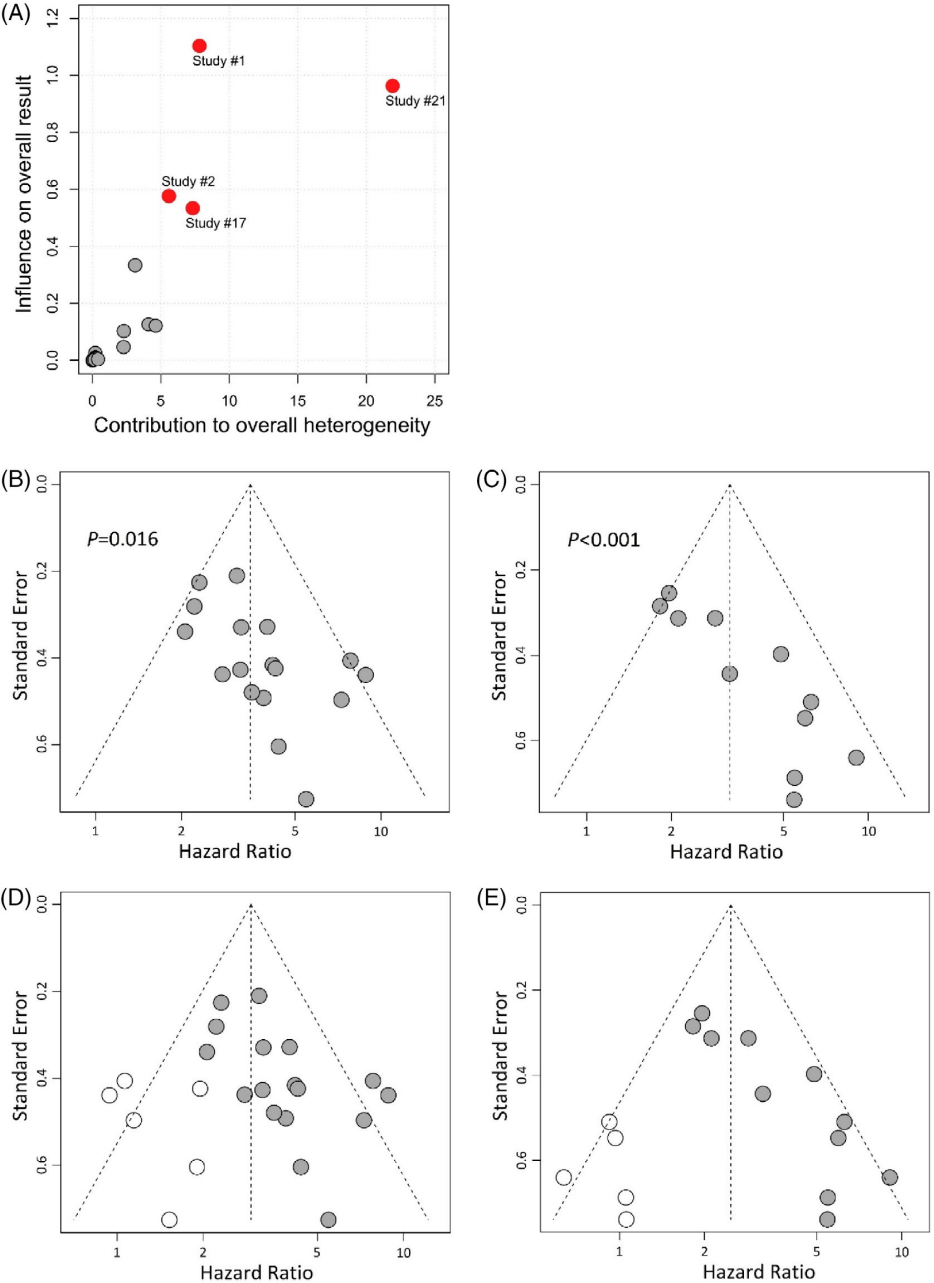
Outliers (A) and funnel plots for publication bias in analyses with post‐*EGFR* for PFS and OS (B and C), and for PFS, OS after imputing missing studies (D and E). OS, overall survival; PFS, progression‐free survival

After treatment with EGFR TKIs (22 studies), *EGFR* clearance in plasma (T + P−) was recorded in a total of 1123 patients, while the persistence or recurrence of this mutation (T + P+) was noted in 500 cases. The median PFS of T + P+ patients was 1.8–11.1 versus 9.8–NR months in T + P− subjects. These OS values in T + P+ and T + P− patients were 7.5–27.0 and 23.7–NR months, respectively. Meta‐analyses have shown that *EGFR*+ in post‐treatment plasma is associated with shorter PFS (HR = 3.84, 95% CI: 2.96–4.99, *p* < .001, Figure 4A) and OS (HR = 3.22, 95% CI: 2.35–4.42, *p* < .001, Figure 4B). While the heterogeneity in OS analysis was relatively low (*I* ^2^ = 39%, *p* = .083), this parameter in the PFS analysis was substantial (*I* ^2^ = 68%, *p* < .001). This phenomenon also was observed in subgroup meta‐analyses (Tables 3 and 4). Subsequently, four studies that contributed most to overall heterogeneity in PFS analysis were detected by influence analysis (Figure 5A). By excluding outliers from the analysis model, the heterogeneity dropped to 22% (*p* = .196), whereas the analyzed result remained significant (HR = 3.49, 95% CI: 2.85–4.27, *p* < .001). Because of the potential publication bias (Figure 5B,C), we used the Trim‐and‐fill statistics to implement missing studies (Figure 5D,E) and showed an adjusted HR value of 2.93 (95% CI: 2.34–3.68, *p* < .001) for PFS, and 2.48 (95% CI: 1.78–3.46, *p* < .001) for OS.

**TABLE 3:** Subgroup meta‐analyses of post‐*EGFR* for PFS

Variable	No. of study	No. of patient	HR (95% CI)	*p*‐Value^*^	Heterogeneity		*p*‐Value^***^
					*I* ^2^, %	*p*‐Value^**^	
Ethnicity							
Asian	12	951	3.85 (2.88–5.15)	<0.001	52	0.018	0.011
Caucasian	7	469	4.75 (2.57–8.78)	<0.001	81	<0.001	
Mixed	2	145	2.02 (1.39–2.93)	<0.001	0	0.943	
Treatment							
1st‐/2nd‐gen TKI	14	1174	4.11 (2.92–5.78)	<0.001	69	<0.001	0.484
Osimertinib	7	391	3.39 (2.24–5.14)	<0.001	67	0.006	
Technique							
ARMS	1	94	3.53 (1.38–9.03)	0.009	‐	‐	0.144
asPCR	3	274	3.46 (2.47–4.84)	<0.001	0	0.712	
dPCR	10	726	4.50 (2.78–7.30)	<0.001	81	<0.001	
PCR clamping	2	247	2.09 (1.46–2.99)	<0.001	0	0.470	
NGS	4	206	3.74 (2.19–6.40)	<0.001	58	0.069	
Other^a^	1	18	4.38 (1.34–14.32)	0.015	‐	‐	
HR extraction method							
Direct	13	1020	3.39 (2.53–4.53)	<0.001	48	0.027	0.359
Indirect	8	545	4.38 (2.75–6.99)	<0.001	80	<0.001	
Survival analysis							
Multivariate	7	574	3.63 (2.37–5.57)	<0.001	67	0.006	0.753
Univariate	14	991	3.97 (2.82–5.58)	<0.001	70	<0.001	
Clinical trial							
No	5	443	3.04 (1.94–4.78)	<0.001	62	0.033	0.271
Yes	16	1122	4.15 (3.03–5.67)	<0.001	69	<0.001	

*Note*: ^*^Significance within groups; ^**^significance of heterogeneity; ^***^significance between groups.

^a^ BEAMing, PANAMutyper, MBP‐QP; 1st‐/2nd‐gen: first‐/second‐generation.

Abbreviations: HR, hazard ratio; NGS, next‐generation sequencing; OS, overall survival; PCR, polymerase chain reaction; TKI, tyrosine kinase inhibitor.

**TABLE 4:** Subgroup meta‐analyses of post‐*EGFR* for OS

Variable	No. of study	No. of patient	HR (95%CI)	*p*‐Value^*^	Heterogeneity			*p*‐Value^***^
					*I* ^2^, %	*p*‐Value^**^		
Ethnicity								
Asian	5	301	2.62 (1.71–4.03)	<0.001	36	0.183		0.093
Caucasian	6	440	3.84 (2.49–5.92)	<0.001	34	0.180		
Treatment								
1st‐/2nd‐gen TKI	8	595	2.80 (2.00–3.92)	<0.001	35	0.149		0.044
Osimertinib	3	146	4.68 (2.77–7.93)	<0.001	0	0.407		
Technique								
asPCR	2	220	3.22 (1.04–9.95)	0.042	76	0.041		0.087
dPCR	5	324	4.30 (2.89–6.42)	<0.001	0	0.458		
PCR clamping	2	120	1.95 (1.29–2.95)	0.002	0	0.723		
NGS	1	59	3.22 (1.35–7.69)	0.008	‐	‐		
Other^a^	1	18	5.48 (1.42–21.09)	0.013	‐	‐		
HR extraction method								
Direct	8	530	3.32 (2.30–4.78)	<0.001	36	0.141		0.973
Indirect	3	211	3.27 (1.49–7.15)	<0.001	62	0.071		
Survival analysis								
Multivariate	5	407	2.61 (1.88–3.63)	<0.001	18	0.302		0.109
Univariate	6	334	4.59 (2.50–8.41)	<0.001	52	0.064		
Clinical trial								
No	4	349	2.82 (1.87–4.26)	<0.001	31	0.226		0.684
Yes	7	392	3.81 (2.31–6.28)	<0.001	50	0.059		

*Note*: ^*^Significance within groups; ^**^significance of heterogeneity; ^***^significance between groups.

^a^ BEAMing, PANAMutyper, MBP‐QP; 1st‐/2nd‐gen: first‐/second‐generation.

Abbreviations: HR, hazard ratio; NGS, next‐generation sequencing; OS, overall survival; PCR, polymerase chain reaction; TKI, tyrosine kinase inhibitor.

## 4 DISCUSSION

Several studies have been conducted to assess the prognostic role of the *EGFR*‐plasma test in NSCLC treated with EGFR TKIs, however, with different conclusions.^3^, ^4^, ^5^, ^6^, ^7^, ^8^, ^9^, ^10^, ^11^, ^12^, ^13^, ^14^ Thus, it has not been recommended to use in prognosis yet.^1^ We performed the meta‐analysis on *EGFR* positive tumor NSCLC from 35 studies and noted that *EGFR*+ in both tumor tissue and plasma at baseline is the worse prognostic factor for PFS and OS. Additionally, the maintained detectable *EGFR* (*EGFR* ^E19del^ and *EGFR* ^L858R^ ± *EGFR* ^T790M^) or recurrence of the mutation in plasma after EGFR TKI initiation is the inferior factor for survival outcomes. Significantly, the prognosis role of the *EGFR*‐plasma test is also validated in treatment with third‐generation EGFR TKI, and for different technique as PCR clamping, allele‐specific PCR, digital PCR, and NGS.

Patients with plasma concurrent *EGFR* mutations are classified into the shedding tumor group and associated with poor performance status, advanced clinical stage, increased metastatic site, and large tumor volume.^7^, ^8^, ^42^ In addition, *EGFR* plasma concomitance is correlated with a higher percentage of driver mutations and gene alterations (*TP53, CDK4/6, CTNNB1, AR, PIK3CA, MYC, CCNE1, KRAS, PDGFRA, NF1…*).^43^ It explains why the T + P+ patients are less sensitive to EGFR TKIs and have shorter survival compared to those with non‐shedding *EGFR* mutations. Moreover, baseline *EGFR*‐plasma and coexisting alterations are related to the mutation persisting in post‐treatment samples^28^ and the development of secondary mutations as *EGFR* ^T790M^, *EGFR* ^C797S^, and other acquired genetic changes.^43^, ^44^, ^45^ These are consistent with the meta‐analyzed results that maintenance of initial *EGFR* mutations (with or without secondary mutations) in plasma is the worse signature. Thanks to the benefit of prognosis, clinicians should require additional *EGFR*‐plasma mutation testing even though it has been confirmed positive in the tumor tissues. Also, bi‐monthly repeated monitoring of *EGFR* mutations in plasma after EGFR TKI initiation should be done in NSCLC management.

This study highlights the prognostic role of the *EGFR*‐plasma test in NSCLC treated with EGFR TKIs. However, some limitations still exist. First, substantial heterogeneity and publication bias is present in post‐treatment analyses, although non‐trial studies without adjusted HR values have been excluded. It might be due to differences in patient characteristics, therapy regimen, and HR extraction method between studies. Thus, cautious use of results is needed. Second, the sample size in some study arms is limited, while not all individual HR values are extracted directly, which might affect the overall results. Third, this study only finishes with the prognostic role of *EGFR*‐plasma as a single gene, which requires further clinical trials with a complex gene model to continue to update our results.

In conclusion, the results of this study indicated that NSCLC patients harboring *EGFR‐*plasma mutations have poorer outcomes compared to those with tumor‐only mutations during EGFR TKI therapies. Besides, the persistence of *EGFR* mutations in post‐treatment plasma is the worse factor for PFS and OS.

## CONFLICT OF INTEREST

The authors declare no conflict of interest.

## AUTHOR CONTRIBUTIONS

*Conceptualization, data curation, formal analysis, investigation, methodology, software, supervision, validation, writing—original draft, writing—review and editing*, T.P.; *Data curation, formal analysis, investigation, resources, validation, writing—review and editing*, V.T.; *Data curation, formal analysis, investigation, validation, writing—original draft, writing—review and editing*, B.‐T.T.; *Data curation, formal analysis, investigation, resources, validation, visualization, writing—review and editing*, T.H.; *Data curation, investigation, validation, writing—review and editing*, S.P.; *Investigation, validation, writing—review and editing*, V.L.; *Data curation, investigation, validation, writing—review and editing*, A.L.; *Data curation, investigation, validation, writing—review and editing*, H.N.; *Conceptualization, data curation, formal analysis, investigation, methodology, project administration, resources, supervision, validation, writing—review and editing*, S.N.

## ETHICAL STATEMENT

Not applicable.

## Supporting information


**Table S1** Characteristics of studies included in the meta‐analysis

## Data Availability

Data sharing is not applicable to this article as no new data were created or analyzed in this study.
