# Supplementary material for: EGFR ‐plasma mutations in prognosis for non‐small cell lung cancer treated with EGFR TKIs: A meta‐analysis
Source: Cancer Rep (Hoboken). 2021 Aug 23;5(8):e1544. doi: 10.1002/cnr2.1544 (PMC9351650; doi:10.1002/cnr2.1544)
Supplement: Supplementary file 1 — Table S1 Characteristics of studies included in the meta‐analysis [file CNR2-5-e1544-s001.docx]

**Table S1.** Characteristics of studies included in the meta-analysis

| **Author** | **Year** | **Country** | **Ethnicity** | **Study design** | **N**  **(T+P+/T+P-)** | **Median age** | **Clinical stage** | **Treatment** | **Sampling** | **Technique** | **Follow-up (months)** | **Outcome** | **Survival analysis** | **HR extraction method** | **NOS** | **Ref.** |
| --- | --- | --- | --- | --- | --- | --- | --- | --- | --- | --- | --- | --- | --- | --- | --- | --- |
| Rosell R | 2009 | Spain | Caucasian | Pro | 164 (97/67) | 67 | IIIB-IV | Erl | S/Prior | PCR clamping | 14 | PFS, OS | M | D | 8 | 19 |
| Mok T | 2015 | Asian | Asian | T | 122 (42/80) | 59 | III-IV | Erl+chemo | P/Post | asPCR | 28.2 | PFS, OS | U | I | 9 | 20 |
| Tseng JS | 2015 | Taiwan | Asian | Pro | 62 (9/53) | 64 | IIIB-IV | Erl/Gef | P/Post | PCR clamping | NA | PFS, OS | M | D | 7 | 21 |
| Lee JY | 2016 | Korea | Asian | T | 40 (6/34) | 58 | IV | Erl/Gef | P/Post | dPCR | 18.8 | PFS, OS | U | D | 8 | 22 |
| Oxnard GR | 2016 | Global | Mixed | T | 104 (69/35) | 61 | III-IV | Osi, 2^nd^ line | P/Prior | BEAMing | 26.3 | PFS | U | I | 8 | 5 |
| Sueoka-Aragane N | 2016 | Japan | Asian | T | 87 (35/52) | 68 | II-IV | Erl/Gef | P/Prior | MBP-QP | NA | OS | U | I | 7 | 23 |
| Kim CG | 2017 | Korea | Asian | Pro | 102 (70/32) | 61 | IIIB-IV | Erl/Gef | P/Prior | PANAMutyper | 36.4 | PFS, OS | M | D | 8 | 24 |
|  |  |  |  |  | 18 (10/8) |  |  |  | P/Post |  |  |  |  |  |  |  |
| Mok T | 2017 | Global | Mixed | T | 124 (100/24) | 60 | III-IV | Gef+chemo | P/Prior | BEAMing | PFS:11.2 | PFS, OS | U | I | 9 | 6 |
| Wu YL£ | 2017 | Global | Mixed | T | 171 (50/121) | 61 | IIIB-IV | Afa, 1^st^ line | S/Prior | asPCR | PFS:16.4 | PFS, OS | U | I | 9 | 7 |
| Wu YL$ | 2017 | Asian | Asian | T | 207 (135/72) | 58 | IIIB-IV | Afa, 1^st^ line | P/Prior | asPCR | PFS:16.6 | PFS, OS | U | I | 9 | 7 |
| Lee Y | 2018 | Korea | Asian | T | 57 (34/23) | 57 | IIIB-IV | Erl/Gef/Afa | P/Prior | PANAMutyper | 23.3 | PFS | M | D | 8 | 25 |
| Shepherd FA | 2018 | Global | Mixed | T | 129 (48/81) | 62 | IIIB-IV | Osi, 2^nd^ line | P/Post | dPCR | NA | PFS | U | D | 7 | 26 |
| Taus Á | 2018 | Spain | Caucasian | T | 33 (11/22) | 70 | III-IV | Gef/Afa/chemo | P/Post | dPCR | NA | PFS | U | I | 6 | 27 |
| Wang Z | 2018 | China | Asian | T | 167 (20/147) | 57 | IV | Gef | P/Post | dPCR | 14.5 | PFS | U | I | 8 | 28 |
| Wu YL¥ | 2018 | Asian | Asian | T | 147 (95/52) | 61 | IIIB-IV | Erl | P/Prior | asPCR | 25.6 | PFS, OS | U | I | 9 | 8 |
| Wu YL§ | 2018 | Asian | Asian | T | 49 (38/11) | 59 | IIIB-IV | Erl+chemo | P/Prior | asPCR | 28.2 | PFS, OS | U | I | 9 | 8 |
| Akamatsu H | 2019 | Japan | Asian | T | 55 (34/21) | 69 | IIIB-IV | Afa, 1^st^ line | P/Prior | dPCR | 14.2 | PFS | U | I | 9 | 9 |
|  |  |  |  |  | 33 (13/20) |  |  |  | P/Post |  |  |  |  |  |  |  |
| Bordi P | 2019 | Italy | Caucasian | T | 38 (32/6) | 65 | IIIB-IV | Osi, 2^nd^ line | P/Prior | dPCR | 33 | PFS, OS | U | I | 8 | 29 |
| Ding PN | 2019 | Australia | Caucasian | T | 26 (5/21) | 67 | IIIB-IV | Erl/Gef | P/Post | dPCR | 18 | PFS, OS | U | D | 8 | 30 |
| Gray JE | 2019 | Global | Mixed | T | 243 (183/60) | 64 | IIIB-IV | Osi, 1^st^ line | P/Prior | asPCR | 16.2 | PFS | U | I | 9 | 10 |
| Phan TT | 2019 | Vietnam | Asian | Pro | 33 (16/17) | 61 | IV | Erl/Gef±chemo | P/Prior | ARMS | NA | OS | M | D | 6 | 31 |
|  |  |  |  |  | 94 (57/37) |  |  |  | P/Post |  |  | PFS |  |  |  |  |
| Wu YL | 2019 | China | Asian | T | 85 (55/30) | 57 | IIIB-IV | Erl | P/Prior | asPCR | 28.9 | PFS, OS | U | I | 8 | 11 |
| Buder A | 2020 | Austria | Caucasian | Pro | 108 (58/50) | 69 | IV | Osi, 2^nd^ line | P/Prior | dPCR | 32.3 | PFS, OS | M | D | 9 | 32 |
|  |  |  |  |  | 57 (19/38) |  |  |  | P/Post |  |  |  |  |  |  |  |
| Ebert EBF† | 2020 | Denmark | Caucasian | T | 142 (98/44) | 67 | IIIB-IV | Erl | P/Prior | asPCR | 16.7 | PFS, OS | U | D | 9 | 12 |
|  |  |  |  |  | 98 (21/77) |  |  |  | P/Post |  |  |  |  |  |  |  |
| Ebert EBF* | 2020 | Denmark | Caucasian | T | 54 (20/34) | 67 | IIIB-IV | Osi, 2^nd^ line | P/Post | asPCR | 10.5 | PFS | U | D | 8 | 33 |
| Fukuhara T | 2020 | Japan | Asian | T | 215 (147/68) | 75 | IIIB-IV | Erl+Bev | P/Prior | PCR clamping | 12.4 | PFS | U | D | 9 | 13 |
|  |  |  |  |  | 185 (22/163) |  |  |  | P/Post |  |  |  |  |  |  |  |
| Iwama E | 2020 | Japan | Asian | T | 84 (47/37) | 69 | IIIB-IV | Erl/Gef/Afa/Osi | P/Prior | dPCR | 16.3 | PFS | U | D | 9 | 34 |
|  |  |  |  |  | 40 (32/8) |  |  |  | P/Post |  |  |  |  |  |  |  |
| Molina-Vila MA | 2020 | Europe | Caucasian | T | 91 (55/36) | 66 | IIIB-IV | Erl+Bev | P,S/Prior | PCR clamping | 52.5 | PFS | M | D | 9 | 14 |
|  |  |  |  |  | 58 (29/29) |  |  |  | P,S/Post |  |  | OS |  |  |  |  |
| Pender A | 2020 | Canada | Caucasian | Ret | 177 (76/101) | 66 | IIIB-IV | Erl/Gef/Afa/Osi | P/Prior | dPCR | 13.2 | OS | M | D | 7 | 35 |
| Yu HA‡ | 2020 | USA | Caucasian | T | 30 (7/23) | 60 | IV | Osi, 1^st^ line+Bev | P/Post | dPCR | NA | PFS, OS | U | I | 7 | 36 |
| Ai X | 2021 | China | Asian | T | 85 (16/69) | 61 | IIIB-IV | Erl/Gef/Ico/Afa/Osi | P/Post | NGS | 10.0 | PFS | M | I | 8 | 37 |
| Ma L | 2021 | China | Asian | Pro | 59 (24/35) | 64 | IIIB-IV | Osi, 2^nd^ line | P/Post | NGS | 43 | PFS, OS | M | I | 8 | 38 |
| Provencio M | 2021 | Spain | Caucasian | Pro | 171 (68/103) | 67 | IV | Erl/Gef/Afa/Osi | P/Post | dPCR | 28 | PFS, OS | M | D | 7 | 39 |
| Sakai K | 2021 | Japan | Asian | T | 46 (13/33) | 67 | III-IV | Osi, 2^nd^ line | P/Post | NGS | 12 | PFS | M | D | 8 | 40 |
| Yu HA# | 2021 | Global | Mixed | T | 16 (8/8) | 64 | I-IV | Osi, 2^nd^ line+Ram | P/Post | NGS | 25 | PFS | U | I | 8 | 41 |

**Abbreviations:** T+P+: *EGFR*+ in both tumor tissue and plasma/serum; T+P-: *EGFR*+ in tumor tissue but not in plasma/serum; Erl: erlotinib; Gef: gefitinib; Afa: afatinib; Ico: icotinib; Osi: osimertinib; Bev: bevacizumab; Ram: ramucirumab; Chemo: chemotherapy; NA: not available; P/Prior: plasma/prior-treatment; P/Post: plasma/post-treatment; S/Prior: serum/prior-treatment; S/Post: serum/post-treatment; PFS: progression-free survival; OS: overall survival; U: univariate; M: multivariate; I: indirect; D: direct; NOS: Newcastle-Ottawa scale; asPCR: allele-specific polymerase chain reaction; dPCR: digital polymerase chain reaction; NGS: next generation sequencing; BEAMing: beads, emulsion, amplification, magnetics; MBP-QP: mutation-biased PCR and quenching probe; Ref.: reference; T: trial; Pro: prospective study; Ret: retrospective study.

£: LUX-Lung 3 study; $: LUX-Lung 6 study; ¥: ASPIRATION study; §: FASTACT-2 study; †: NCT02284633, Erl; *: NCT02284633, Osi 2^nd^ line; #: NCT02789345; ‡: NCT02803203.
